# Limitations and perspectives of the novel salivary test for endometriosis: an open web-based survey study of German gynecologic healthcare providers

**DOI:** 10.1007/s00404-024-07601-3

**Published:** 2024-09-26

**Authors:** Meletios P. Nigdelis, Merle Doerk, Stefanie Burghaus, Martin Sillem, Bashar Haj Hamoud, Erich-Franz Solomayer, Gregor Leonhard Olmes

**Affiliations:** 1 Department of Gynecology, Obstetrics and Reproductive Medicine, Saarland University Hospital, Kirrberger Straße 100, Building 9, 66421 Homburg, Saarland, Germany; 2 Department of Gynecology and Obstetrics, University Endometriosis Center for Franconia, Erlangen University Hospital, Friedrich Alexander University of Erlangen-Nuremberg, Universitätsstrasse 21–23, 91054 Erlangen, Germany

**Keywords:** Endometriosis, Salivary test, miRNA, Non-invasive diagnosis

## Abstract

**Introduction:**

The description of a salivary miRNA signature for endometriosis has led to the development of a non-invasive diagnostic test. Current healthcare provider practices regarding the test remain uncaptured. The application of this test in practice was examined in a web-based survey, with the aim to provide their opinions on it.

**Methods:**

We conducted an open web-based survey study between November 2023 and January 2024. Members of the German society of gynecologic endoscopy (Arbeitsgemeinschaft gynäkologische Endoskopie, AGE), society of endometriosis (Arbeitsgemeinschaft Endometriose, AGEM), and the endometriosis research foundation (Stiftung Endometriose Forschung, SEF) were contacted per e-mail twice. Participants’ data were anonymized. Differences in responses based on self-reported expertise in the field (basic knowledge, specialized knowledge, expert) were assessed using the χ^2^-test or Fisher’s exact test. Statistical significance was set as *p* < 0.05.

**Results:**

In total 141 of 190 respondents completely responded to the survey (> 75% of the questions of the survey). Twenty-one physicians reported having experience with the test, while most participants had at least specialized knowledge on the field (112/141). In terms of specific questions, more than 90% found the costs high; almost 85% did not believe that the test replaces standard diagnostic tools (histology, clinical examination, and sonography). Eighty-six providers supported the use of the test in adolescents. Gynecologists with basic knowledge had a more positive attitude compared with more experienced ones in terms of usefulness (Fisher’s exact test, *p* < 0.001). Significant differences were demonstrated between expertise groups regarding (not only) applicability in adolescents (Fisher’s exact test, *p* = 0.004), and using the test for screening purposes (χ^2^-test, *p* = 0.002).

**Discussion:**

Despite the promising benefits of a salivary test for endometriosis, German healthcare providers would not change current practices. Nevertheless, less experienced colleagues were more positive towards the test.

**Supplementary Information:**

The online version contains supplementary material available at 10.1007/s00404-024-07601-3.

## What does this study add to the clinical work

**Table:** 

A small number of survey participants (14.8%) reported having experience with the salivary test. German healthcare providers would most probably not change current clinical practice, even though the test could be used in specific circumstances (e.g. adolescents). This opinion was more common among less experienced providers compared to more experience ones.	

## Introduction

Endometriosis is estimated to affect as many as 10% of female-born people, leading to almost 190 million cases worldwide [1, 2]. Individuals with the disorder face a multifaced detrimental health burden, not only because of consecutive interventions, for example multiple surgeries or reproductive technologies, but also non-reproductive sequelae, such as chronic pain, poorer mental health and quality of life, cardiovascular disease, and cancer compared with the general population [3]. This is reflected not only on a personal level, but also on a societal level with significant consequences for healthcare systems as well [4, 5]. According to an analysis by Simoens and colleagues, the annual costs for women with the disease reached €9579 (95% confidence interval €8559 to €10,599) on average, approximately those of patients with diabetes or Crohn’s disease [6].

One of the severe challenges in endometriosis, recognized in the 2023 Endometriosis Fact Sheet by the world health organization (WHO), pertains to its diagnosis, specifically the diagnostic delay between symptoms and treatment onset [7, 8]. Diagnosis of the disease should be based on history taking, signs of disease, clinical examination, and imaging studies (ultrasound and/or magnetic resonance imaging), while the previous gold standard of laparoscopy is no longer required in all cases, as noted in national and international guidelines [9, 10]. Recently, advances in precision medicine, artificial intelligence, and omics-based technologies have led to the identification of disease-specific biomarkers in blood, eutopic endometrium and saliva, with some currently undergoing investigation in multicenter trials [11]. Even though these markers might, in theory, offer a non-invasive, specific, and sensitive diagnostic method, current evidence does not support their use [12].

Among these biomarkers, special interest has been focused on microRNA (miRNAs). These molecules constitute non-coding RNAs of 21–25 nucleotides that regulate gene expression by binding to their complementary messenger RNA (mRNA) [13]. In a literature review between 2000 and 2020, 18 studies were identified evaluating miRNA expression levels in patients with endometriosis and controls. According to the study, 63 miRNAs were differentially expressed in the blood of women with the disease, of which only 14 yielded replicable findings in other studies. Of note, in some studies panels of miRNAs provided diagnostic accuracy measures comparable to or better than those of laparoscopy, which further explains the great interest surrounding miRNAs in the diagnosis of endometriosis [14].

In a recent study (ENDO-miRNA) by Bendifallah et al*.* salivary miRNAs were prospectively assessed in terms of diagnostic accuracy in 200 patients presenting with chronic pelvic pain with or without endometriosis. After genome-wide miRNA expression profiling and bioinformatic analysis of all samples, the authors were able to provide a signature of 109 distinct miRNAs for endometriosis. In terms of diagnostic accuracy, this reached 96.7%, 100%, and 98.3% for sensitivity, specificity, and area under the curve (AUC), respectively [15]. The results of this analysis are currently being validated in a multicenter validation study in France (target population 1000 individuals). In the most recent interim analysis of 200 individuals, the authors could duplicate the diagnostic accuracy measures of the previous study, namely sensitivity 96.2%, specificity 95.1%, positive predictive value (PPV) 95.1%, negative predictive value (NPV), and AUC of 0.96 [16]. Given these significant results, the company Ziwig introduced a promising, efficient, non-invasive salivary test (Endotest^®^) to the market in 2022 [17, 18]. The test quickly gained interest in public. Scientific associations discussed on possible restrictions connected to use [19, 20].

To this day, no survey on healthcare providers has been conducted, assessing their views and experiences on the salivary test. In this web-based questionnaire study we aimed to document the views, opinions, and experiences of German gynecology healthcare providers on the salivary test for endometriosis.

## Materials and methods

This was a web-based open cross-sectional survey conducted between November 2023 and January 2024. The survey was conducted using the online software LimeSurvey (https://www.limesurvey.org/, ID: 817,746). All responses were anonymized. Moreover, we used an integrated cookie method provided by the software to prevent repeated participation in the survey. The survey comprised of 18 questions (see **Supplementary File 1**) in the German language. The first part was related to baseline information of the participants (‘*Allgemeine Informationen*’, see **Supplementary File 1**), while the second part consisted of questions specific to the salivary test (‘*Fragen zum Speicheltest*’, see **Supplementary File 1**).

Registered members of the German society of endometriosis (Arbeitsgemeinschaft Endometriose, AGEM), the endometriosis research foundation (Stiftung Endometriose Forschung, SEF), and the German society of gynecologic endoscopy (Arbeitsgemeinschaft gynäkologische Endoskopie, AGE)-in this case all emails registered to the newsletter of the society-, were contacted through each society per email twice (November and December 2023). The email included a link to the online survey. Furthermore, the survey was advertised during the 15th Congress of Endometriosis for German speaking countries (November 23–25, 2023, in Saarbrücken, Germany).

As far as the number of members is concerned, there are currently 170 AGEM members, 116 SEF members, and 2370 AGE members-approximately 5,000 email addresses are registered to the newsletter. Due to significant overlap of members between all three societies, along with inactive memberships (e.g. colleagues no longer practicing in Germany), an exact estimation of the recipients was not possible.

### Ethics and reporting guidelines

The manuscript abides by the Consensus-Based Checklist for Reporting of Survey Studies (CROSS) Guidelines for reporting of survey studies, as suggested by the equator network (see **Supplementary Table 1**) [21]. Furthermore, official approval was provided by the bioethics committee of the medical association of Saarland (Registration number 213/23).

### Statistical analysis

Data were extracted in a Microsoft Excel (Version 16.82) file for further analysis. Descriptive statistics in the form of absolute counts (percentage) were calculated for every survey item.

Regarding calculation of response rate, this was based on the following principle, given that the exact calculation of recipients was not possible (see above). A total of 190 respondents accessed and completed questions of the survey. From them, 49 (25.8%) responded to no question pertaining to the salivary test (second part of the survey, see above) (incomplete responders), while the rest (74.2%) responded to at least 75% of the survey items (complete response rate). 

Group differences, based on self-reported experience level in the field of endometriosis, were examined using the chi-squared test or Fisher’s exact test in case where values for a specific group were < 5. Statistical significance was defined as *p* < 0.05. We undertook no measures for missing data, as this did not exceed 5%. The statistical program Jamovi (2.3.21.0) was used for the analysis.

## Results

General characteristics of the participants along with non-responses are summarized in Table 1, while Table 2 summarizes the responses (along with non-responses) of participants to questions pertaining to salivary test, itself.

**Table 1:** General characteristics of the survey participants

Variable	*N* = 141 Count (%)
Work position	
Resident (Arzt/ Ärtzin in Weiterbildung)	9 (6.4%)
Specialist (Facharzt/ärztin)	9 (6.4%)
Attending physician (Oberarzt/ärztin)	72 (51.1%)
Chief physician (Chefarzt/ärztin)	30 (21.3%)
Private practice	20 (14.2%)
No response	1 (0.7%)
Work experience (years)	
0–5 years	7 (5.0%)
5–10 years	19 (13.5%)
10–15 years	35 (24.8%)
> 15 years	80 (56.7%)
No response	–
Work environment	
Hospital (endometriosis certified facility)	31 (22.0%)
Hospital (not certified endometriosis facility)	60 (42.6%)
University hospital	29 (20.6%)
Practice	17 (12.1%)
No active clinical duty (e.g. maternity leave, retirement, research rotation)	4 (2.8%)
No response	–
Experience in the field of endometriosis (self-reported)	
Basic knowledge	29 (20.6%)
Specialized knowledge	48 (34.0%)
Expert	64 (45.4%)
No response	-
Number of tests you have conducted	
0	118 (83.7%)
Less than 5	16 (11.3%)
5–25	5 (3.5%)
No response	2 (1.4%)

**Table 2:** Responses to specific questions of the survey pertaining to the salivary test.

Variable	N = 141 Count (%)
How do you find the costs (about 800 Euros) of salivary test?	
Affordable	1 (0.7%)
Cost-neutral	4 (2.8%)
Expensive	46 (32.6%)
Too expensive	89 (63.1%)
No response	1 (0.7%)
The costs of the test were covered	
By the patients themselves	17 (12.1%)
By the insurance provider (after application)	9 (6.4%)
No response	115 (81.6%)
The salivary test replaces the	
Histological confirmation	17 (12.1%)
Sonography	2 (1.4%)
Clinical examination	3 (2.1%)
None of the options mentioned above	119 (84.4%)
In your opinion, how do you find the test as a new diagnostic tool?	
Indispensable	3 (2.1%)
A meaningful addition	30 (21.3%)
Usable in specific instances	70 (49.6%)
Dispensable	36 (25.5%)
No response	2 (1.4%)
In your opinion, can the salivary test be applied as a screening method for endometriosis?	
Yes	41 (29.1%)
No	97 (68.8%)
No response	3 (2.1%)
In your opinion, can the saliva test be used to confirm the diagnosis of endometriosis?	
Yes	58 (41.1%)
No	82 (58.2%)
No response	1 (0.7%)
Would you conduct the test in an adolescent patient?	
Yes	86 (61.0%)
No	54 (38.3%)
No response	1 (0.7%)
If not, why not?	
Lack of evidence and validation for individuals under 18 years of age	34 (24.1%)
Risk of overdiagnosis	25 (17.7%)
Lack of interpretability of the test result	33 (23.4%)
I would conduct the test	
In every patient with a suspicion of endometriosis	18 (12.8%)
Only if sonography or MRI are negative for endometriosis	68 (48.2%)
Not at all	54 (38.3%)
No response	1 (0.7%)
When would you conduct the test?	
Initially for diagnostic confirmation	40 (28.4%)
Before a laparoscopy	35 (24.8%)
Before initiating medical therapy	33 (23.4%)
When there is suspicion of an endometriosis recurrence	35 (24.8%)
Before initiating fertility treatment	39 (27.7%)
A patient with dysmenorrhea comes to you with a positive test result. What is your approach?	
Initiation of medical therapy (e.g. dienogest 2 mg)	76 (53.9%)
Conducting an MRI of the pelvis	2 (1.4%)
In any case, laparoscopic surgery	11 (7.8%)
Laparoscopy in case of pronounced symptoms	48 (34.0%)
No response	4 (2.8%)
A patient with endometriosis symptoms (e.g., dysmenorrhea) and a normal ultrasound result comes to you with a negative saliva test result. What is your approach?	
Initiation of medical therapy (e.g. dienogest 2 mg)	57 (40.4%)
Conducting an MRI of the pelvis	12 (8.5%)
In any case, laparoscopic surgery	4 (2.8%)
Laparoscopy in case of pronounced symptoms	64 (45.4%)
No response	4 (2.8%)
In your opinion, how satisfied are the patients with the test?	
Not satisfied at all	1 (0.7%)
Not satisfied	2 (1.4%)
Neutral	13 (9.2%)
Satisfied	7 (5.0%)
Very satisfied	5 (3.5%)
No information provided, as I haven't had any experience with the test so far	110 (78.0%)
No responses	3 (2.1%)

*MRI* magnetic resonance imaging

Our cohort comprised primarily of gynecologists who have completed specialty training (78.8%; working as specialist, attending, chief positions), with at least 5 years working experience (95%), reporting at least specialized knowledge on the field of endometriosis (79.4%). A total of 21 physicians (14.8%) reported having conducted the salivary test.

In terms of usability, 30 physicians considered the test a meaningful addition to clinical routine (21.3%), while 70 (49.6%) would use it only under specific circumstances. We investigated this aspect further and demonstrated that 68 (48.2%) participants would consider the test in case imaging findings turned out negative. Despite this fact, 119 (84.4%) did not believe, that the test replaces any of the standard diagnostic tools of endometriosis (histology, ultrasound, clinical examination). Another aspect raised by the survey, was the opinions on costs. Almost 64% of participants considered the costs of the test extremely high. Regarding cost coverage, 9 (6.4%) physicians reported coverage by the insurance provider after proper application, while 17 (12.1%) providers reported self-coverage of the costs by the patients.

In terms of the applying of the test in the adolescent population, 86 participants (61%) agreed with its usage. Among those disagreeing with the use of the test, 34 based their choice, due to lack of evidence/validation for individuals under 18 years of age, 33 considered a lack of interpretability of the test results, while 25 supported that the test might lead to overdiagnosis.

Two of the questions in our survey were clinical scenarios which had to do with patients presenting with symptoms suggestive of endometriosis and a) a positive test result, and b) negative test with a negative ultrasonography for endometriosis. In the first scenario, most clinicians [76 (53.9%)] would initiate medical therapy for endometriosis, while in the second, most participants would perform a laparoscopy in case of pronounced symptoms [64 (45.4%)] or initiate medical therapy [57 (40.4%)].

Table 3 demonstrates differences in the responses of participants based on reported expertise level on endometriosis. Statistically significant differences were demonstrated in questions including but not limited to the use of the test in adolescents (Fisher’s exact test, p = 0.004), applicability of the test as a screening tool (χ^2^-test, p = 0.002), and its costs (Fisher’s test, p = 0.037).

**Table 3:** Comparison of responses among participants with different expertise level on endometriosis.

	Basic knowledge *n* = 29 (20.6%)	Specialized knowledge *n* = 48 (34.0%)	Expert *n* = 64 (45.4%)	*p*-value
How do you find the costs (about 800 Euros) of salivary test?				**0.037**^**1**^
Affordable	0 (0.0%)	1 (2.1%)	0 (0.0%)	
Cost-neutral	1 (3.4%)	2 (4.2%)	1 (1.6%)	
Expensive	16 (55.2%)	13 (27.1%)	17 (27.0%)	
Too expensive	12 (41.4%)	32 (66.7%)	45 (71.4%)	
The salivary test replaces the				
Histological confirmation (yes/no)	4 (13.8%)	8 (16.7%)	5 (7.8%)	0.386^1^
Sonography (yes/no)	0 (0%)	1 (2.1%)	1 (1.6%)	1.000^1^
Clinical examination (yes/no)	0 (0%)	2 (4.2%)	1 (1.6%)	0.594^1^
None of the options mentioned above (yes/no)	24 (82.8%)	39 (81.3%)	56 (87.5%)	0.641^2^
In your opinion, how do you find the test as a new diagnostic tool?				< **0.001**^1^
Indispensable	0 (0%)	2 (4.3%)	1 (1.6%)	
A meaningful addition	14 (48.3%)	7 (14.9%)	9 (14.3%)	
Usable in specific instances	14 (48.3%)	29 (61.7%)	27 (42.9%)	
Dispensable	1 (3.4%)	9 (19.1%)	26 (41.3%)	
In your opinion, can the salivary test be applied as a screening method for endometriosis?				**0.002**^2^
Yes	16 (57.1%)	11 (23.4%)	14 (22.2%)	
No	12 (42.9%)	36 (76.6%)	49 (77.8%)	
Would you conduct the test in an adolescent patient?				**0.004**^1^
Yes	25 (86.2%)	29 (60.4%)	32 (50.8%)	
No	4 (13.8%)	19 (39.6%)	31 (49.2%)	
I would conduct the test				< **0.001**^1^
In every patient with a suspicion of endometriosis	8 (27.6%)	5 (10.4%)	5 (7.9%)	
Only if sonography or MRI are negative for endometriosis	17 (58.6%)	29 (60.4%)	22 (34.9%)	
Not at all	4 (13.8%)	14 (29.2%)	36 (57.1%)	
When would you conduct the test?				
Initially for diagnostic confirmation (yes/no)	11 (37.9%)	14 (29.2%)	15 (23.4%)	0.352^2^
Before a laparoscopy (yes/no)	13 (44.8%)	13 (27.1%)	9 (14.1%)	**0.006**^**2**^
Before initiating medical therapy (yes/no)	9 (31.0%)	12 (25.0%)	12 (18.8%)	0.410^2^
When there is suspicion of an endometriosis recurrence (yes/no)	13 (44.8%)	14 (29.2%)	8 (12.5%)	**0.003**^**2**^
Before initiating fertility treatment (yes/no)	10 (34.5%)	19 (39.6%)	10 (15.6%)	**0.013**^**2**^
A patient with dysmenorrhea comes to you with a positive test result. What is your approach?				0.234^1^
Initiation of medical therapy (e.g. dienogest 2 mg)	12 (41.4%)	25 (54.3%)	39 (62.9%)	
Conducting an MRI of the pelvis	1 (3.4%)	1 (2.2%)	0 (0.0%)	
In any case, laparoscopic surgery	3 (10.3%)	2 (4.3%)	6 (9.7%)	
Laparoscopy in case of pronounced symptoms	13 (44.8%)	18 (39.1%)	17 (27.4%)	
A patient with endometriosis symptoms (e.g., dysmenorrhea) and a normal ultrasound result comes to you with a negative saliva test result. What is your approach?				< **0.001**^1^
Initiation of medical therapy (e.g. dienogest 2 mg)	5 (17.2%)	15 (32.6%)	37 (59.7%)	
Conducting an MRI of the pelvis	6 (20.7%)	5 (10.9%)	1 (1.6%)	
In any case, laparoscopic surgery	1 (3.4%)	1 (2.2%)	2 (3.2%)	
Laparoscopy in case of pronounced symptoms	17 (58.6%)	25 (54.3%)	22 (35.5%)	

Responders with missing responses (see Table 2) were excluded from the statistical test.

Statistically significant results are demonstrated as bold numbers

*MRI* magnetic resonance imaging

^1^p-values represent those of Fisher’s exact test

^2^p-values represent those of the χ^2^-test

## Discussion

Our study demonstrated frontiers and barriers of gynecology providers on the salivary test for endometriosis. Most physicians (85%) agreed that the test cannot replace the value of already established tools, namely histological examination (as part of laparoscopy), clinical examination, and ultrasound. Another important aspect raised by the survey were the costs of the test, which were considered too high by most participants. Moreover, the test should not be considered a screening tool of asymptomatic patients according to almost 70% of the respondents. Furthermore, 61% of physicians responded positively to the question of whether they would conduct the test in adolescents. We were able to demonstrate statistically significant differences in the responses based on self-reported level of experience in the field. In summary, these results suggest that providers with basic knowledge, to some extent younger colleagues, are more in favor of the test compared with providers with specialized knowledge or experts of the field. Finally, most physicians agreed that they would initiate medical treatment in case of symptoms suggestive of endometriosis, independent of the result of the test.

One of the major issues addressed by our study was that of the cost of the test. Most healthcare providers assessed the costs of the salivary test as high. The issue of cost automatically raises questions regarding the ‘optimal’ population, for which the test should be used without imposing significant costs to the patients and healthcare system (cost effectiveness). In an analysis based on the French healthcare system, routine care was compared with the following strategies: 1) all patients receive the test, 2) patients with negative sonography (for endometrioma) receive the test, 3) patients with a negative ultrasound and MRI examination receive the test. At a price of €750, the costs per accurately diagnosed case were €1542 (routine care), €990 (strategy 1), €919 (strategy 2) and €1000 (strategy 3). On the contrary, a lower price per test (e.g. €500) rendered routine care the most cost-effective [22]. Despite this perspective, real-world data on the economic burden of the test are lacking. Further considering that in some countries, like Germany, the cost is not covered by the insurance companies leaves open questions, which need to be addressed in the future.

Another important topic requiring attention is the applicability of the test in different subpopulations based on scientific evidence. First, it has been demonstrated that miRNAomes are characterized by significant differences in different populations and ethnicities [23, 24]. This aspect has not been addressed in the analyses by Bendifallah et al*.*, which did not provide any evidence on the ethnicity of the patients tested [15, 16]. To what extent, the miRNA signature applies to populations outside of France remains to be investigated [20]. Moreover, the necessity of external validation cannot be more stressed, especially when it comes to specific phenotypes such as endometriosis-associated infertility, which was recently addressed in a subanalysis of the original population of the study [25]. Advocating optimal diagnostic accuracy without validation studies hampers the applicability of the evidence. Of note, the introduction of the test to the market was undertaken without published data of an external validation study, an interim analysis of which appeared recently [16].

Using the test for screening purposes (in asymptomatic patients) was discussed in our analysis. Based on the registered protocol of the ENDO-miRNA study (ClinicalTrials.gov NCT04728152), use of miRNA analysis was confined to symptomatic patients treated in tertiary centers while asymptomatic patients were not included [26]. Hence, applying the test in the general population needs to be avoided. Moreover, current evidence pertains to a prospective evaluation, which according to Saquib et al*.* differs from the gold standard to establish screening test efficacy, the randomized controlled trial [27]. Adding to this, Vercellini et al*.* challenged the necessity of a screening tool for endometriosis. The authors argued that even though early detection of the disease is of significance, there is no evidence supporting that early treatment hampers disease progression to the classical phenotype of the disease. Furthermore, the question of optimal management in case of a positive result also remains unanswered and controversial. On a theoretical level, even though some individuals would benefit from screening, others would face the consequences of overdiagnosis and treatment, along with an increased emotional burden [28].

Adolescent patients also received interest in the survey. In our study, 61% of the participants reported, that they would recommend the test to an adolescent girl. We speculate that this view is based on the necessity of a non-invasive diagnostic test for young adolescent patients. Brosens et al*.* provided evidence on adolescent disease progression and presence of severe disease at a young age necessitating timely diagnosis [29]. Despite these important observations, the studies published on the salivary test (to-date) excluded minors, thus, posing questions of external validity in this subpopulation. From an everyday perspective, clinical routine would most probably remain unchanged. Imaging (i.e. ultrasound and/or MRI) would still be required in cases of ‘supposed’ severe disease (e.g. exclusion of hydronephrosis); the test does not differentiate between superficial and deep endometriosis. In terms of therapy, adolescents would begin medical therapy (e.g., suppression of ovulation) along with analgesics, trying to avoid unnecessary surgery, which leaves the exact ‘value’ of the test unclear [30]. Further studies should provide more evidence on adolescents.

Our study presents strengths and limitations. To our knowledge, there has been no published survey for the application of the salivary test in the literature, which makes our findings novel. We have included responses from more than 100 healthcare workers involved in the treatment of patients with endometriosis (75% of the respondents reported being experts). This subgroup of physicians is more likely to have knowledge and experience with the test, making them the most appropriate survey group.

Regarding limitations, clinical and research experience with the salivary test in Germany was limited compared with France. Furthermore, size calculation of our sample entails restrictions. The primary reason for this was the great overlap of the members in the societies involved. Due to data protection rights, direct access to the mailing lists of the societies was not possible; something which would have allowed exact calculation of the response rate. Importantly, we have accounted for multiple participation as mentioned in the methods section. Web-based physician surveys generally demonstrate low response rates as explained by Cunningham et al*.* [31]. Nevertheless physicians constitute a much more homogenous group compared with the general population, given similar education, training, and behaviors [32].

## Conclusion

Our open web-based survey study demonstrated that German gynecologists would not change their diagnostic and therapeutic practice based on current evidence on the salivary test. Nevertheless, the promising benefits of the test as a non-invasive diagnostic method finds given the great need of an accurate, not delayed diagnosis. Less experienced colleagues demonstrated different attitudes compared with more experience ones.

## Supplementary Information

Below is the link to the electronic supplementary material.

Supplementary file1 (DOCX 23 KB)
